# γ-Radiation Enhanced Luminescence of Thiol-Capped Quantum Dots in Aqueous Solution

**DOI:** 10.3390/nano9040506

**Published:** 2019-04-02

**Authors:** Shuquan Chang, Xian Wu, Jianzhang Lan, Zheng Li, Xiaohong Zhang, Haiqian Zhang

**Affiliations:** Jiangsu Engineering Laboratory of Nuclear Energy Equipment Materials, College of Material Science and Technology, Nanjing University of Aeronautics and Astronautics, Nanjing 210016, China; ygihk2012@163.com (X.W.); LJZ9678@163.com (J.L.); lizheng1506@163.com (Z.L.); zhangxiaohong@nuaa.edu.cn (X.Z.); zhanghq@nuaa.edu.cn (H.Z.)

**Keywords:** quantum dots, luminescence enhancement, γ-radiation, thiol-capped

## Abstract

Quantum dots (QDs) have attracted great attention due to their unique optical properties. High fluorescence efficiency is very important for their practical application. In this study, we report a simple and efficient strategy to enhance the photoluminescence of water-dispersed thiol-capped QDs using γ-radiation. Three kinds of QDs with different surface ligands and cores (MPA-CdTe, MPA-CdSe and Cys-CdTe) were fabricated and irradiated by high-energy γ-ray in an aqueous solution. Their photoluminescence intensities were significantly enhanced after irradiation, which were closely related to the radiation dose and the structure of QDs. The positions of the fluorescence emission peaks did not shift obviously after irradiation. The mechanism of photoluminescence enhancement was discussed based on the results of photoluminescence (PL) spectra, UV-visible light absorption (UV-vis) spectra, transmission electron microscope (TEM), X-ray diffraction (XRD) patterns, Fourier transform infrared (FT-IR) spectra and X-ray photoelectron spectroscopy (XPS). This method can be employed to uniformly treat large batches of QDs at room temperature and without other chemicals.

## 1. Introduction

Colloid semiconductor nanocrystals, which are also known as quantum dots (QDs), exhibit unique optical and optoelectronic properties due to quantum confinement effects [1]. They have attracted great attention for their wide range of applications including biological imaging, medical diagnosis, photodynamic therapy, light-emitting diodes, photovoltaic cells and display devices [2,3,4,5]. High fluorescence efficiency is required for these practical applications. In past decades, efforts have been made to synthesize high-quality QDs and improve their luminescent performance [6,7]. The organometallic synthetic route has become one of the popular methods to prepare highly luminescent II-VI QDs [8]. However, the resulting QDs are insoluble in water, which limits their biomedical applications. When the QDs are transferred from organic solvent into water via further surface modification, their fluorescence efficiency is usually decreased because of surface imperfections. Water-dispersed QDs can also be directly synthesized in an aqueous medium using thiols as stabilizing agents such as thioglycolic acid (TGA), mercaptopropionic acid (MPA) and cysteamine (Cys) [9]. Nevertheless, the as-prepared QDs using this method have lower fluorescence quantum yields comparing with the above organometallic synthetic route, because of the surface defects or charge traps of nanocrystals [10]. One effective strategy to reduce surface traps and enhance the fluorescence efficiency is to epitaxially grow an inorganic shell with a larger band-gap around the core of QDs to form a core–shell structure [1,11]. A series of core–shell QDs such as CdTe/CdS, CdTe/CdSe, CdSe/CdS, CdTe/ZnS and CdTe/CdS/ZnO have been successfully fabricated and developed [11,12,13]. Highly luminescent ZnSe/ZnSe(S) QDs were synthesized using a microwave-assisted method [14]. It has been demonstrated that visible light and UV illumination can affect the structure of QDs, which can be used to enhance the fluorescent efficiency of QDs [15,16]. Bao et al. observed a strong photoluminescence enhancement effect of CdTe QDs stabilized by TGA under room light and concluded that the fluorescence quantum yields reached the maximum in 20 days [17]. Wang et al. found that the photoluminescence of citrate-stabilized CdSe and CdSe/CdS nanocrystals was enhanced after illumination with visible light for 15 days [18]. Zhang et al. reported the concentration-dependent photoluminescence enhancement of PbS QDs, which remained during storage [19]. Valentyn et al. studied the photoactivation of CdS nanocrystals under different conditions and found that the main factor was the adsorption of water molecules on the surface of nanocrystals [20]. Zare et al. reported high-efficiency CdTe/CdS nanocrystals by growing CdS shells on CdTe cores under UV light [21]. This method is considered to be an alternative way to achieve high quality QDs. However, the energy and penetration abilities of UV and visible light are low, which leads to a long activation time and low homogeneity. It cannot be used to treat large quantities of samples in big containers. 

A gamma ray is a kind of ionizing radiation with high energy and a strong penetration ability, which can rapidly trigger homogeneous chemical reactions in the solution via direct or indirect routes at room temperature. Until now, γ-radiation has been widely applied to process traditional materials and synthesize new materials such as metal nanoparticles, semiconductor nanocrystals, hydrogels and anion-exchange membranes, etc. [22,23,24,25,26]. We have successfully fabricated many kinds of novel materials including biocompatible, chitosan-coated ZnS, silk fibroin-coated CdSe, Prussian blue nanocomposites and Ag/MIL-101(Cr) MOFs, etc., via a one-step γ-radiation route [27,28,29,30]. The effects of radiation on the photoluminescence of QDs has attracted attention. Withers et al. and Stodilka et al. reported the degradation of CdSe/ZnS colloidal QDs in hexanes under Cs-137 and Co-60 γ-ray, respectively [31,32]. Gaur et al. observed the reversible photodarkening properties of sub-monolayer CdTe/CdS QDs in porous silica scaffolds under irradiation [33]. Jovanović et al. reported the enhanced photoluminescence of graphene QDs by gamma irradiation [34]. Recently, Zanazzi et al. reported the photoluminescence enhancement of colloidal CdSe/ZnS QDs in polyvinyl alcohol, and the decreased photoluminescence of InGaP/ZnS in polydimethylsiloxane under proton irradiation, and found that the QD embedding medium played an important role in the irradiated optical response [35,36]. We note that the change in photoluminescence (enhancement or attenuation) of QDs after ionizing irradiation is not necessarily the same under different conditions, and may depend on the structure of the QDs, environmental factors and radiation parameters. 

In this paper, we report a simple and efficient strategy to enhance the photoluminescence of water-dispersed, thiol-capped QDs (MPA-CdTe, MPA-CdSe and Cys-CdTe) using γ-radiation. The photoluminescence properties and structures of QDs before and after irradiation were characterized and analyzed using photoluminescence (PL) spectra, UV-visible light absorption (UV-vis) spectra, transmission electron microscope (TEM), X-ray diffraction (XRD) patterns, Fourier transform infrared (FT-IR) spectra and X-ray photoelectron spectroscopy (XPS). The results revealed that the photoluminescence of as-prepared QDs with different particle sizes and surface ligands was obviously enhanced after irradiation, and was closely related to the radiation dose. The mechanism of γ-radiation-induced QD photoluminescence enhancement is discussed in detail. The advantage of this method is that it can be used to uniformly treat large quantities of QD solution at room temperature and without other chemicals. 

## 2. Materials and Methods 

### 2.1. Materials

Cadmium chloride (CdCl_2_·2.5H_2_O, AR, Aladdin), tellurium powder (Te, AR, Sigma), selenium powder (Se, AR, Aladdin), sodium borohydride (NaBH_4_, AR, Macklin), sodium hydroxide (NaOH, AR, Macklin), hydrochloric acid (HCl, AR, Sinopharm), 3-mercaptopropionic acid (MPA, AR, Mackli), cysteamine (Cys, AR, Macklin), isopropyl alcohol (AR, Aladdin), and acetone (AR, Aladdin) were used. All chemicals were used without any further purification. Double distilled water was used in the experiments.

### 2.2. Preparation of MPA-CdTe, MPA-CdSe and Cys-CdTe QDs

In a typical synthesis of MPA-CdTe QDs, 0.12 g NaBH_4_ was dissolved in 2 mL double distilled water with an ice bath while stirring under N_2_ protection, then 0.125 g Te powder was added to the above solution and mixed well to form a NaHTe solution. A total of 0.537 g CdCl_2_·2.5H_2_O and 0.4 mL MPA were dissolved in 125 mL distilled water one by one, then the solution was adjusted to pH = 11 using 1 M NaOH solution and heated to boil. The previously prepared NaHTe solution was added into the boiling solution while stirring strongly, and the mixed solution was heated to reflux under N_2_ protection. MPA-CdTe QDs of different sizes were obtained by taking solution from the bottle at different times. MPA-CdSe and Cys-CdTe QDs were prepared using a similar method as described above using Se powder and cysteamine ligands, respectively. The molar ratios of NaBH_4_:Te/Se:CdCl_2_:MPA/Cys are 3:1:2.5:5 in a typical synthesis. The as-prepared QDs were precipitated and washed with isopropyl alcohol and acetone by centrifugation three times. 

### 2.3. Irradiation of MPA-CdTe, MPA-CdSe and Cys-CdTe QDs in Solution

MPA-CdTe, MPA-CdSe and Cys-CdTe QDs were irradiated in the aqueous solution. Typically, the solution containing QDs was put into a glass bottle and sealed well. The bottle was put into the Co-60 γ-ray equipment (Dose rate: 0.69 kGy·h^−1^) and irradiated at room temperature for a period of time to reach different doses (1, 5 and 10 kGy). After that, the QDs solution was taken out of the radiation equipment. 

### 2.4. Characterization

The morphology, structures and photoluminescence properties of QDs were characterized in detail. Transmission electron microscope (TEM) images were taken using a FEI Tecnai G20 high-resolution transmission electron microscope (FEI, Hillsboro, OR, USA). X-ray diffraction (XRD) patterns were recorded on a BRUKER D8 Advance diffractometer (BRUKER, Karlsruhe, Germany). Fourier transform infrared (FT-IR) spectra were obtained on a BRUKER OPUS 80V FT-IR spectrometer (BRUKER, Karlsruhe, Germany). X-ray photoelectron spectroscopy (XPS) spectra were obtained on a ULVCA PHI-5000 analyzer (ULVCA-PHI, Kanagawa, Japan). Photoluminescence (PL) spectra were taken with a HITACHI 850 spectrophotometer (HITACHI, Tokyo, Japan). UV-visible absorption (UV-vis) spectra were carried out using a PerkinElmer-spectrophotometer (Perkin-Elmer, Waltham, MA, USA). The fluorescence quantum yields (QY) of QDs were measured using Rhodamin 6G as a fluorescence standard based on the following equation: QY_s_ = (F_s_/F_r_) × (A_r_/A_s_) × QY_r_, where F and A were the measured fluorescence (area under the emission peak) and the absorbance at the excitation wavelength, respectively. The optical photos of aqueous solutions containing QDs were taken under room light and 365 nm UV light respectively. 

## 3. Results and Discussion

### 3.1. Photoluminescence Properties of As-Prepared QDs Before and After Irradiation

**Figure 1:**
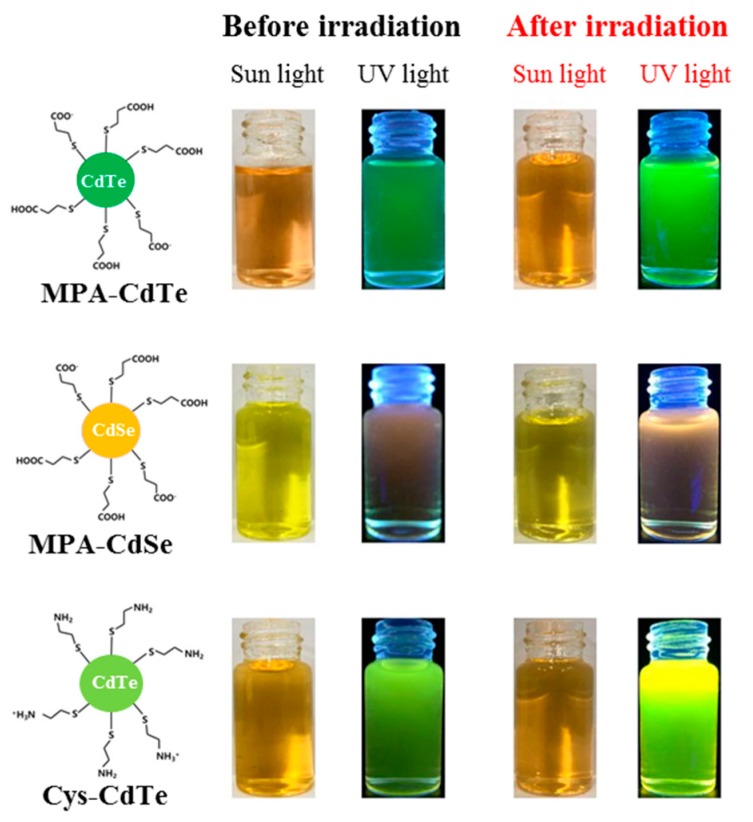
Structural diagrams and optical photos of as-prepared quantum dots (QDs) under different conditions.

**Figure 2:**
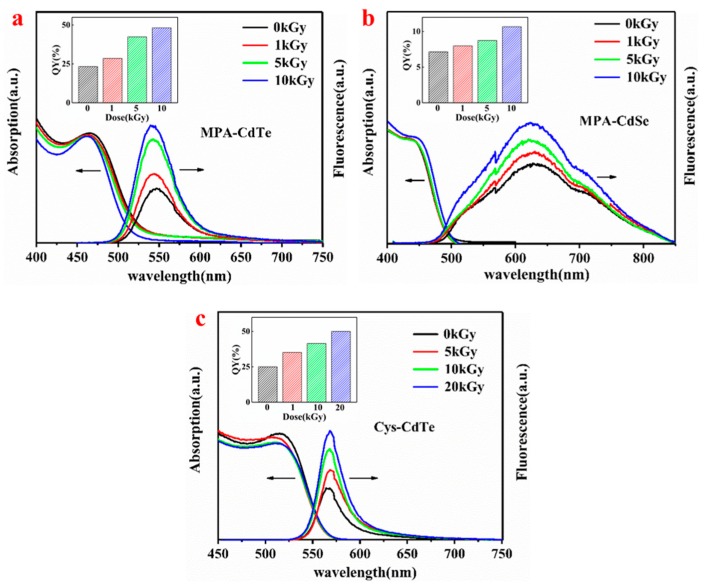
UV-vis, photoluminescence (PL) spectra and quantum yield of MPA-CdTe (**a**), MPA-CdSe (**b**) and Cys-CdTe (**c**) QDs under different radiation doses.

In order to comparatively investigate γ-radiation-induced photoluminescence enhancement, three kinds of water-dispersed thiol-capped QDs with different compositions and surface ligands were employed in this study. The structural diagrams of these are shown in Figure 1. MPA and Cys ligands were coated on the surface of QDs via the thiol group, which not only prevents the aggregation of nanoparticles but also reduces their surface defects to some extent. Figure 1 shows an optical photo of three kinds of QDs in the aqueous solution before and after γ-irradiation under UV and sunlight, respectively. All solutions containing QDs are very transparent, which indicates that the as-prepared QDs have excellent water-dispersivity and the γ-irradiation did not induce their aggregation. The color of QD solutions under sunlight was not obviously changed after γ-irradiation. The as-prepared QDs emitted green, yellow-green and orange fluorescence under UV light. After γ-irradiation, the fluorescence of the QDs under UV light was distinctly enhanced. The fluorescence enhancements of MPA-CdTe and Cys-CdTe QDs were more obvious than MPA-CdSe QDs. In order to quantitatively investigate the fluorescent evolution of QDs after γ-irradiation, the PL spectra were tested in detail and are shown in Figure 2. The fluorescence emission peaks of MPA-CdTe, MPA-CdSe and Cys-CdTe QDs were around 545 nm, 626 nm and 565 nm, respectively. The positions of the above fluorescence emission peaks and UV-vis absorption peaks did not shift obviously after irradiation, which indicates that there was no obvious change in particle size. The fluorescence intensities of the three kinds of QDs were gradually enhanced as the γ-radiation dose increased. The fluorescence QYs of MPA-CdTe, MPA-CdSe and Cys-CdTe QDs were 23.3%, 7.1% and 25% before irradiation, respectively. They increased to 48.2%, 10.7% and 41.5% after 10 kGy irradiation, respectively. The fluorescence QY of Cys-CdTe QDs reached 50% when the radiation dose was 20 kGy. These results are consistent with the optical photos under UV light. 

**Figure 3:**
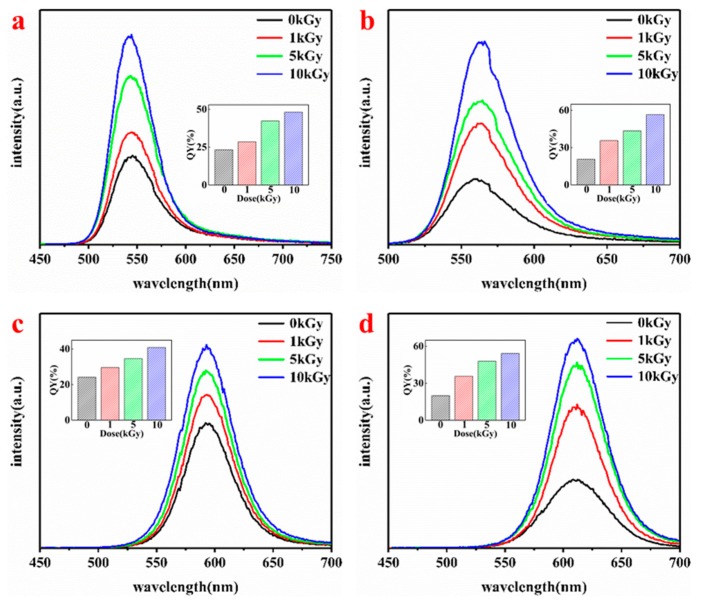
PL spectra and quantum yield of MPA-CdTe QDs with different particle sizes (QDs-545 (**a**), QDs-567 (**b**), QDs-592 (**c**) and QDs-608 (**d**)) after γ-irradiation.

The photoluminescence properties of QDs are closely related to their particle size. Therefore, the PL spectra of MPA-CdTe QDs with different particle sizes after different dose radiations were tested and are shown in Figure 3. The particle sizes of the MPA-CdTe QDs in Figure 3a–d were about 3.4 nm, 3.6 nm, 4.0 nm and 4.3 nm, and were obtained with a reaction time of 0.5 h, 0.75 h, 2 h and 3 h, respectively. The fluorescence emission peaks of the four kinds MPA-CdTe QDs were around 545 nm, 567 nm, 592 nm and 608 nm. The positions of their fluorescence emission peaks did not change obviously after irradiation. Their fluorescence intensities were gradually enhanced as the γ-radiation dose increased. After 10 kGy γ-irradiation, the fluorescence QYs of QDs-545, QDs-567, QDs-592 and QDs-608 increased respectively to 48.2%, 56.6%, 40.8%, and 54.2%; these results are 207%, 275%, 169% and 272% of their original QYs before irradiation. These results indicate that there was no definite relationship between the irradiation-induced fluorescence enhancement efficiency and the particle size of the QDs.

### 3.2. Morphology and Structure of As-Prepared QDs Before and After γ-Irradiation

**Figure 4:**
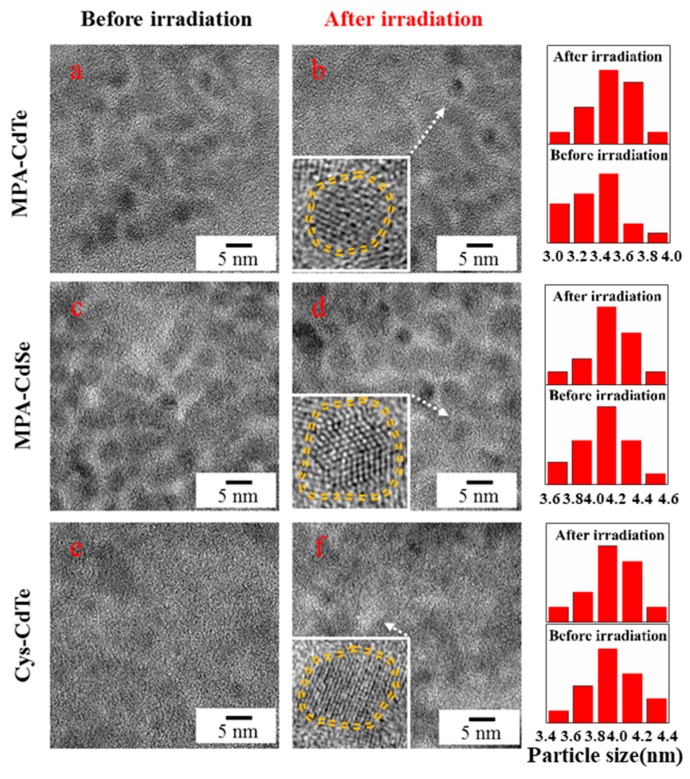
TEM images and size distributions of MPA-CdTe (**a**,**b**), MPA-CdSe (**c**,**d**) and Cys-CdTe (**e**,**f**) QDsbefore and after 10 kGy γ-irradiation.

**Figure 5:**
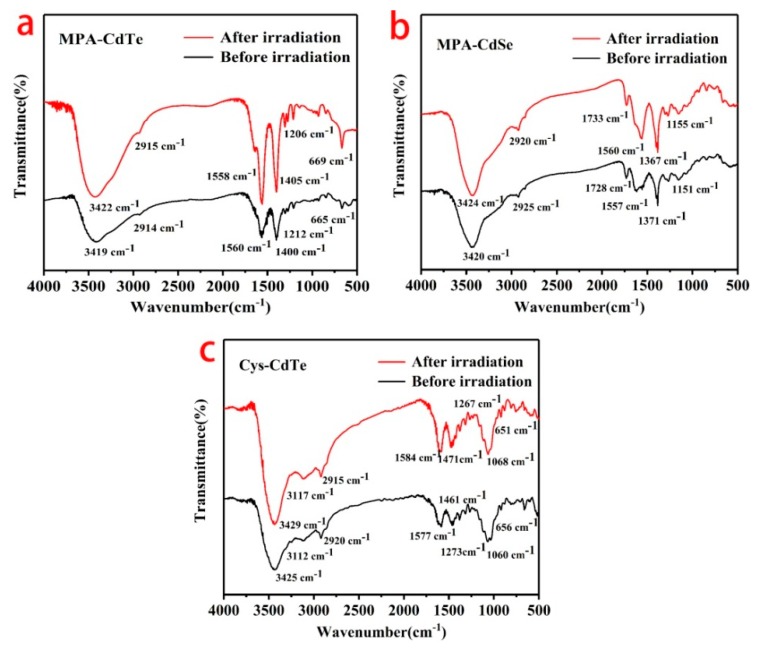
FTIR spectra of MPA-CdTe (**a**), MPA-CdSe (**b**) and Cys-CdTe (**c**) QDs before and after 10 kGy γ-irradiation.

Micrographs of three samples before and after γ-irradiation were characterized using TEM and are presented in Figure 4. The as-prepared QDs had an approximately spherical shape and were well dispersed. The core sizes of MPA-CdTe, MPA-CdSe and Cys-CdTe QDs were about 3.4 nm, 4.1 nm and 3.9 nm, respectively, which was similar to the samples after irradiation. The γ-radiation did not induce significant changes to the core sizes of the QDs. The insets in Figure 4 reveal that the crystal structures of the core were not damaged by irradiation and thin shells coated the core of the QDs. As shown in the HRTEM results, the thickness of the shell around the QDs was about 0.4–0.7 nm in these samples. The shells were well capped on the surface of the MPA-CdTe and Cys-CdTe cores. The shell around MPA-CdSe was not very complete. FTIR spectra of three samples ranging from 500 cm^−1^ and 4000 cm^−1^ were tested to investigate the changes of the surface ligands on the QDs before and after γ-irradiation. As is shown in Figure 5a, the peaks of the MPA-CdTe QDs at around 3419 cm^−1^, 2914 cm^−1^, 1560 cm^−1^, 1400 cm^−1^, and 665 cm^−1^ were ascribed to υ (OH), υ (CH_2_), υ (C=O), υ (C-O), and δ (C-S), which are the characteristic peaks of MPA [10]. The S-H vibration (around 2550 cm^−1^) was not detectable in the spectra of the MPA-CdTe QDs, which was expected for thiols covalently bound to the surface of the QDs. The characteristic peaks of the MPA-CdTe QDs did not change obviously after γ-irradiation. Similar results were observed in the FTIR spectra of the MPA-CdSe and Cys-CdTe QDs, which indicates that the surface ligands of the QDs were not damaged seriously by irradiation under these conditions. 

**Figure 6:**
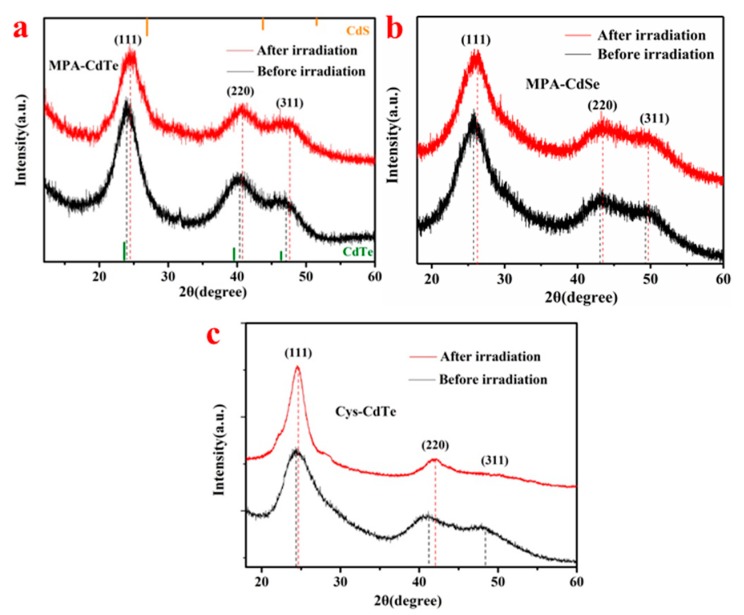
XRD patterns of MPA-CdTe (**a**), MPA-CdSe (**b**) and Cys-CdTe (**c**) QDs before and after 10 kGy γ-irradiation.

The XRD patterns of three samples before and after γ-irradiation are shown in Figure 6. There are three obvious peaks in the XRD pattern of MPA-CdTe QDs at around 24.1°, 40.3°and 47.1°, which can be assigned to the (111), (220) and (311) planes of cubic CdTe (JCPDS 15-0770), respectively [10,17]. After γ-irradiation, the above diffraction peaks shifted slightly to larger angles at around 24.6°, 40.7°and 47.6°, respectively. The characteristic peaks of the CdS crystals were around 26.8°, 43.7°and 52.1° (JCPDS 42-1411) [37]. The shift of the diffraction peaks toward the peaks of the cubic CdS crystals indicates the formation of CdS on the surface of the CdTe core [17]. The characteristic peaks of cubic CdSe crystals are at around 25.2°, 41.1°and 48.2° (JCPDS 19-0191) [27]. As is shown in Figure 6b, the diffraction peaks of the MPA-CdSe QDs also shifted slightly toward the peaks of the cubic CdS crystals after γ-irradiation, which also indicated the formation of crystalline CdS on CdSe. The XRD pattern of Cys-CdTe QDs showed a similar tendency to that of the MPA-CdTe QDs. It has been reported that fabricating an inorganic shell with a larger band-gap around the core of the QDs can enhance their fluorescence efficiency [11]. The formation of crystalline CdS shells on CdTe and CdSe cores under γ-irradiation will reduce defect sites and hence trap states, which might be an important cause of photoluminescence enhancement.

**Figure 7:**
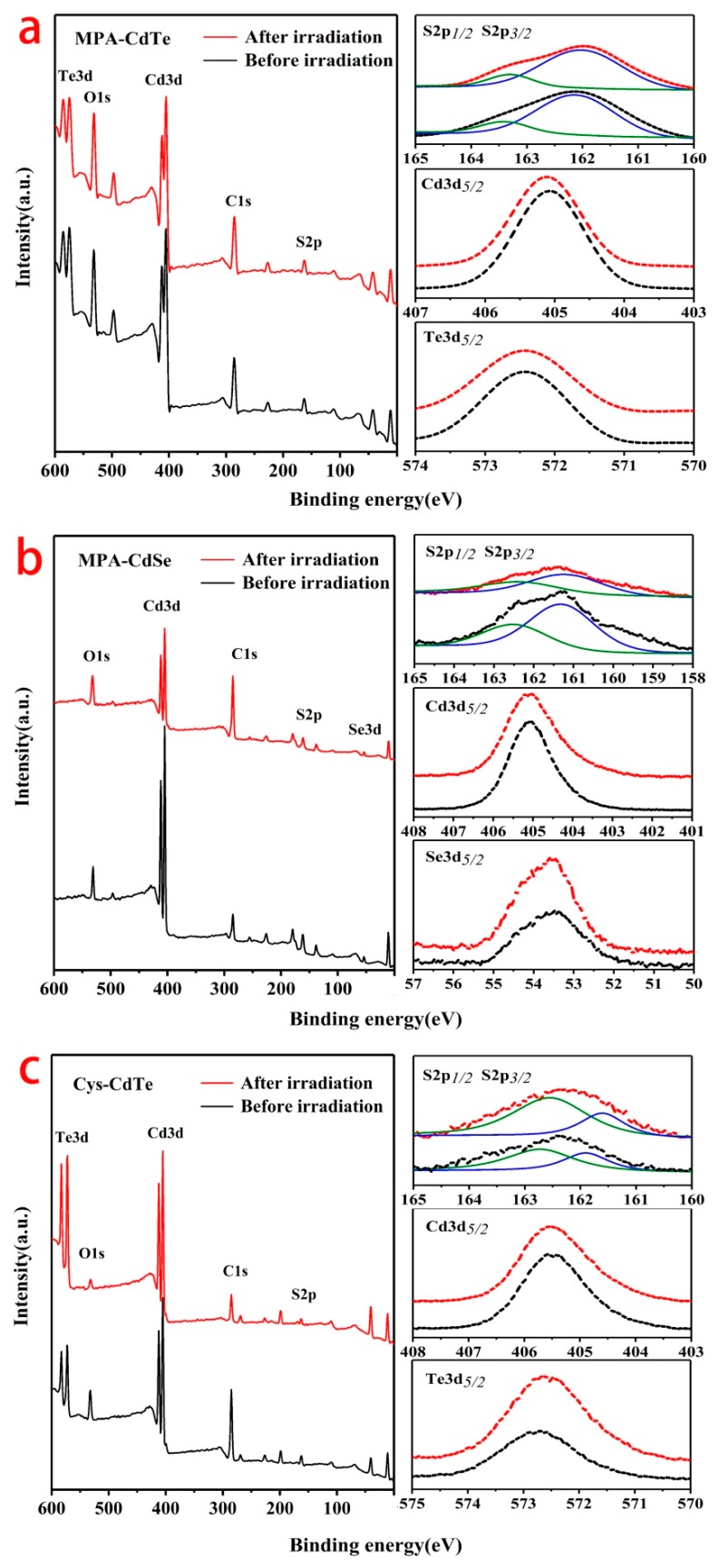
XPS spectra of MPA-CdTe (**a**), MPA-CdSe (**b**) and Cys-CdTe (**c**) QDs before and after 10 kGy irradiation.

The XPS spectra were tested to demonstrate the changes on the surface of the QDs under γ-irradiation. Figure 7 shows the XPS survey spectra and high-resolution spectra for S2p, Cd3d, Te3d, Se3d of MPA-CdTe, MPA-CdSe and Cys-CdTe QDs. As shown in Figure 7a, there was no obvious change in the survey spectrum of the MPA-CdTe QDs after γ-irradiation, which indicates that radiation did not significantly change the elemental composition and surface structure of the QDs. However, slight changes were observed on the high-resolution spectra of S2p, Cd3d and Te3d. The S2p spectrum had a typical doublet structure, which could be well-fitted with one doublet of the S2p_1/2_ peak at 163.45 eV and the S2p_3/2_ peak at 162.13 eV. These binding energies correspond to the thiolate between MPA ligands and Cd on the surface of CdTe QDs [17]. After γ-irradiation, the peaks of the S2p spectra slightly shift to lower energy, with an S2p_1/2_ peak at 163.32 eV and an S2p_3/2_ peak at 612.02 eV, which can be attributed to the contribution of S from CdS. The Cd3d_5/2_ peak of MPA-CdTe QDs is positioned at 405.06 eV before irradiation. This peak shifts to a higher binding energy of 405.12 eV after irradiation, which is due to the change of coordination situation with S or Te [21]. The Te3d_5/2_ peak of MPA-CdTe QDs shifts from 512.42 eV to 512.38 eV after γ-irradiation. It has been reported that Te on the surface usually presents a higher binding energy than that in the inner part of CdTe [38]. Thus, the slight decrease in the binding energy of Te3d_5/2_ is attributed to the reduction of Te on the surface of QDs, which also supports the formation of a coating or shell on the surface of QDs under irradiation. The XPS spectra of MPA-CdSe and Cys-CdTe QDs showed a similar tendency to that of MPA-CdTe QDs. The above results reveal the formation of CdS shells on CdTe and CdSe QDs, which is consistent with the data of the XRD patterns. 

### 3.3. Mechanism of γ-Radiation-Induced QD Photoluminescence Enhancement

**Figure 8:**
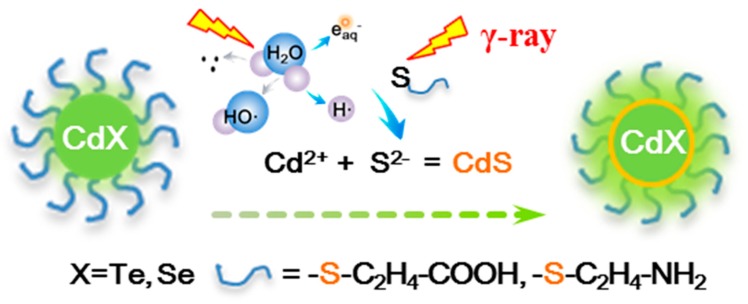
Schematic diagram of the mechanism of γ-radiation-induced luminescence enhancement of thiol-capped QDs in the aqueous solution.

The photoluminescence enhancement of water-dispersed, thiol-capped QDs is mainly attributed to the radiochemical reactions triggered by γ-radiation in aqueous solution. A gamma ray is a high energy photon, which can interact with water molecules, organic ligands (MPA or Cys) and inorganic nanocrystals (CdTe or CdSe) in the solution. A possible reaction mechanism is shown in Figure 8. Water molecules are decomposed under γ-radiation and produce many kinds of active species such as e_aq_^−^, H•, HO• and HO_2_^−^, etc. Reductive active species are dominant in the alkaline solution, which can attack MPA and Cys ligands on the surface of QDs to form S^2−^. These active species interact with the CdTe and CdSe nanocrystals to release Cd^2+^ ions. S^2−^ and Cd^2+^ can also be generated by the direct interaction between γ-radiation and thiol-capped QDs. S^2−^ and Cd^2+^ rapidly combine to form CdS. The release of S^2−^ and Cd^2+^ under γ-radiation is very slow in this reaction system, which leads to the formation of a CdS shell on the surface of QDs rather than CdS particles in the solution. The amount of CdS is not significant, and is limited by the reactants. The thin CdS shells can obviously reduce the surface traps and defects of CdTe and CdSe QDs. The band-gap of CdS is larger than that of CdTe and CdSe. Thus, the formation of thin CdS shell enhances the fluorescence efficiency of thiol-capped QDs, which is considered to be a key factor. 

As mentioned above, UV and visible light can induce chemical reactions on the surface of QDs, such as the oxidation and decomposition of the capping ligand, which can lead to the formation of shells on the surface of a QD core [15,16]. UV/vis illumination has been considered a convenient and feasible way to enhance the fluorescence of QDs [19,20,21]. However, the energy and penetration abilities of UV and visible light are very low, which leads to a long activation time and low homogeneity [17,18]. Compared with light illumination methods, the γ-radiation method proposed in this paper has the following advantages: (1) γ-ray has high energy and can obviously enhance the fluorescence of QDs in a short time; (2) γ-ray has a strong penetration ability and can uniformly trigger chemical reactions in the whole system, which leads to the homogeneity of products; (3) γ-ray can easily penetrate large containers (transparent or opaque), which can be employed to uniformly treat large batches of QDs at one time. Compared with traditional chemical methods, this γ-radiation method is usually carried out at room temperature under ambient pressure in an aqueous solution without excess chemicals, which can reduce the damage to other materials and reduce environmental pollution. 

## 4. Conclusions

A simple and efficient strategy was proposed to enhance the photoluminescence of water-dispersed thiol-capped QDs using γ-radiation. The photoluminescence of QDs with different particle sizes and surface ligands was obviously enhanced after irradiation, and this was closely related to the radiation dose. The position of the fluorescence emission peaks were not obviously changed after irradiation. The photoluminescence enhancement of thiol-capped QDs is mainly attributed to the formation of a thin CdS shell on CdTe and CdSe QDs under γ-irradiation. This method can be employed to uniformly treat large batches of QDs at one time.
